# Unraveling the Potential of *Saccharum officinarum* and *Chlorella vulgaris* towards 5-Fluorouracil-Induced Nephrotoxicity in Rats

**DOI:** 10.3390/ph17070885

**Published:** 2024-07-04

**Authors:** Hanem F. El-Gendy, Amanallah El-Bahrawy, Doaa A. Mansour, Nagwa I. Sheraiba, Nazema S. Abdel-Megeid, Shaimaa Selim, Rashed A. Alhotan, Anam Ayyoub, Saber El Hanbally

**Affiliations:** 1 Department of Pharmacology, Faculty of Veterinary Medicine, University of Sadat City, Sadat City 32897, Egypt; 2 Department of Pathology, Faculty of Veterinary Medicine, University of Sadat City, Sadat City 32897, Egypt; 3 Department of Biochemistry and Chemistry of Nutrition, Faculty of Veterinary Medicine, University of Sadat City, Sadat City 32897, Egypt; 4 Department of Husbandry and Animal Wealth Development, Faculty of Veterinary Medicine, University of Sadat City, Sadat City 32897, Egypt; 5 Department of Cytology and Histology, Faculty of Veterinary Medicine, University of Sadat City, Sadat City 32897, Egypt; 6 Department of Nutrition and Clinical Nutrition, Faculty of Veterinary Medicine, Menoufia University, Shibin El-Kom 32514, Egypt; 7 Department of Animal Production, College of Food and Agriculture Sciences, King Saud University, Riyadh 11451, Saudi Arabia; 8 College of Life Sciences, Northwest A & F University, Yangling District, Xianyang 712100, China

**Keywords:** *Saccharum officinarum*, *Chlorella vulgaris*, 5-fluorouracil, antioxidant, rats, inflammatory cytokine, nephrotoxicity

## Abstract

5-Fluorouracil (5-FU) is often used as a chemotherapeutic agent in treating tumors and is said to have adverse effects, including nephrotoxicity. Therefore, the present study aimed to evaluate the protective effects of *Chlorella vulgaris* (VL) and *Saccharum officinarum* L. (SOL) against 5-FU-induced nephrotoxicity in rats through the measurement of renal histology, kidney damage indicators, and antioxidant measures. A total of forty-eight male rats were allotted into six groups: group 1 acted as a control negative group (control), group 2 received 5-FU and worked as a control positive group (FU), group 3 received SOL 15 mL/kg (SOL), group 4 received VL 400 mg/kg (VL), group 5 received 5-FU+SOL (5-FU+SOL), and group 6 received 5-FU+VL (5-FU+VL). After fifteen days, blood and renal tissue specimens were collected for hematological, biochemical, molecular, and histopathological examinations. Findings of the current investigation showed that 5-FU leads to hematological alterations and kidney injury evinced by elevated serum concentrations of uric acid, creatinine, and urea (*p* < 0.01), and a marked increase in kidney MDA and NO levels with a reduction in kidney CAT, SOD and GSH activities (*p* < 0.05). Alterations of the histopathological structure of kidney tissue in the FU group were noticed compared to the other groups. 5-FU administration elevated expression levels of *TNF-α, lipocalin 2*, and *KIM*1 (*p* < 0.01) compared to the control ones. 5-FU-induced nephrotoxicity was ameliorated after treatment with SOL and VL via their free radical scavenging, potent antioxidant, and anti-inflammatory effects. In conclusion, our findings demonstrate that the treatment with SOL and VL significantly improved nephrotoxicity induced by 5-FU in rats.

## 1. Introduction

Chemotherapeutic drug nephrotoxicity is still a serious side effect that confines their clinical applications. Clinical usage of anticarcinogenic drugs in the campaign against malignancies can lead to a range of renal diseases. Renal impairment has been linked to anticancer drugs such as 5-fluorouracil (5-FU) [1]. 5-Fluorouracil is an analog of pyrimidine uracil and it is a chemotherapy medication that is frequently used to treat a variety of malignant tumors, such as malignancies of the breast, colon, pancreas, skin, stomach, esophagus, and head and neck regions. Administration of 5-FU or other fluoropyrimidines (FPs) has become the standard of care for patients with colorectal cancer [2].

5-FU has a systemic effect as an antitumor agent. 5-FU’s mode of action is based on anabolic processes that produce functional metabolites such as fluoro-deoxyuridine monophosphate, fluoro-deoxyuridine triphosphate), and fluorouridine triphosphate. Fluoro-deoxyuridine monophosphate combines with thymidylate synthase and prevents its function, inhibiting DNA synthesis [3]. These metabolite triphosphates are disincorporated into DNA and RNA, respectively, causing genetic material synthesis to be disrupted and, as a result, cell death. 5-FU also increases mitochondrial reactive oxygen species (ROS) production, into which cytochrome C is generated from mitochondria, causing oxidation [4].

The systemic activity of 5-FU is accompanied by several detrimental impacts that might result in treatment discontinuation, reducing its efficacy. Previously published research showed that the unfavorable influences of 5-FU are concentrated in tissues with a high number of proliferative cells, for instance, the intestines and bone marrow [5,6]. Nevertheless, essential organs with poor proliferative capability, for example, the liver, lungs, and kidney are frequently subjected to 5-FU metabolites and could be adversely damaged. Negative effects have included veno-occlusive disease, steatohepatitis, hepatitis, nephropathy, steatosis, and chest discomfort. These degenerative processes could be triggered by oxidative damage and inflammation caused by 5-FU treatment [7]. Consequently, the search for possible natural constituents to ameliorate the side effects of chemotherapy has regained attention. Therefore, administrating natural antioxidants could be a suitable medicinal approach to lessen 5-FU-induced renal impairment or injury.

Sugarcane (*Saccharum officinarum* L.) (SOL) is a commonly cultivated plant in subtropical and tropical areas, and it is farmed as a significant supplier of sugar. The most profitable portion of the plant from an industrial standpoint is the stem, which comprises comparatively great amounts of sugar. The sugarcane top, juice, has been observed to contain polyphenols with antioxidant properties [8]. The most relevant compounds in sugarcane juice were the flavones diosmetin-8-C-glucoside, vitexin schaftoside, isoschaftoside, and 4′,5′-dimethyl-luteolin-8-C-glucoside, these were detected by HPLC, and these components were reported to induce antioxidant activities [8,9]. Antioxidants and chelating agents found in SOL such as phenolic components are linked to several beneficial impacts on health. The 1,1-diphenyl-2-picrylhydrazyl (DPPH) radical was the target of sugarcane juice’s antioxidant activity. It was observed that flavonoids and phenolic acids, such as ferulic acid and quercitrin, were involved in antioxidant action [10].

*Chlorella vulgaris* (VL) is a single-celled, readily farmed, extremely productive green microalga that is commonly utilized in dietary supplements due to its high nutrient content. As a result, the Food and Drug Administration (FDA) recognized VL as a trustworthy alga for dietary supplementation [11]. It contains high-quality protein (approximately 50–60%), amino acids (18%), vitamins (20%), and minerals such as calcium, iron, magnesium, phosphorus, and potassium [12]. Furthermore, owing to its high concentration of phenolics, carotenoids, lutein, phycobiliproteins, chlorophyll, and astaxanthin, VL exhibits valuable antioxidant and therapeutic characteristics [13]. VL was reported to induce protective effects against organ function disruption through its beneficial physiological effects such as antioxidative, hypocholesterolemic, anti-inflammatory, and immune-stimulant activities. VL was observed to induce hepatoprotective and reno-protective effects in rats and mice [11,14,15].

The goal of the present trial was to investigate the nephroprotective potential of *Saccharum officinarum* L. *juice* and *Chlorella vulgaris* suspension towards 5-FU-induced nephrotoxicity in male albino rats. We hypothesized that VL and SOL could ameliorate the nephrotoxicity induced by 5-FU through their antioxidant and anti-inflammatory properties.

## 2. Results

### 2.1. Body Weight and Relative Organ Weight

In the current trial, a considerable reduction in body weight and gain was observed in the 5-FU group compared to control. However, SOL and VL alone or in combination with 5-FU significantly increased (*p* < 0.01) the weight and weight gain of the rats (Table 1). A noteworthy improvement in the feed conversion compared to the 5-FU-treated group (Table 1). Concerning the relative organ weights, non-significant variations were distinguished among the experimental treatments (Table 2).

### 2.2. Blood Measurements

The effect of administration of SOL and VL alone or with 5-FU-treated rats on blood indices is presented in Table 3. Remarkable (*p* < 0.05) increases were recorded in the RBCs, Hb, and PCV values between the control negative and SOL and VL groups. Conversely, there was a non-significant variation among the SOL+5-FU and the VL+5-FU groups compared to the control rats. On the contrary, the 5-Fu-treated rats exhibited a significant decrease (*p* < 0.05) in the RBCs, PCV, and Hb levels together with a reduction in the mean value of MCV and MCH, without any variations in the values of MCHC compared to those of the control negative. Furthermore, there was an elevation in RDW (*p* < 0.05) in the 5-FU-treated group compared to the other treatment groups. RDW values were decreased in the control negative group (*p* < 0.05). The SOL, VL, and SOL+5-FU, VL+5-FU groups showed significant improvements and reinstated erythrogram indices to the average control ranges. Instead, 5-FU-treated rats exhibited lower values of erythrogram parameters than the normal control values. Concerning the alterations in the leukogram and N/L ratio, data from Table 3 indicated no significant variation among the experimental groups. However, rats intoxicated with 5-FU exhibited a significant decrease (*p* < 0.05) in the counts of TWBCs and lymphocytes, in addition to a rise in N/L ratio compared to those of the other groups (*p* < 0.05).

### 2.3. Kidney Function

Considerable nephrotoxicity was induced by 5-FU compared to control rats, indicated by raised serum creatinine, urea, and uric acid concentrations. No variation in total protein and albumin was observed between the treatment groups. The administration of SOL and VL along with 5-FU significantly reestablished the normal control values compared to the 5-FU group (Table 4).

### 2.4. Kidney Oxidant and Antioxidant Parameters

Serum MDA and NO contents were significantly increased due to the administration of 5-FU to rats (*p* < 0.05) corresponding with a decline in CAT, SOD, and GSH compared to the control group and SOL, VL, SOL+5-FUor VL+5-FU. Administration of SOL and VL with 5-FU eliminated these alterations and overturned the earlier result. Noteworthy, the inclusion of the algae mitigates the adverse effect of 5-FU on kidney function (Table 5).

### 2.5. Gene Expression

**Figure 1:**
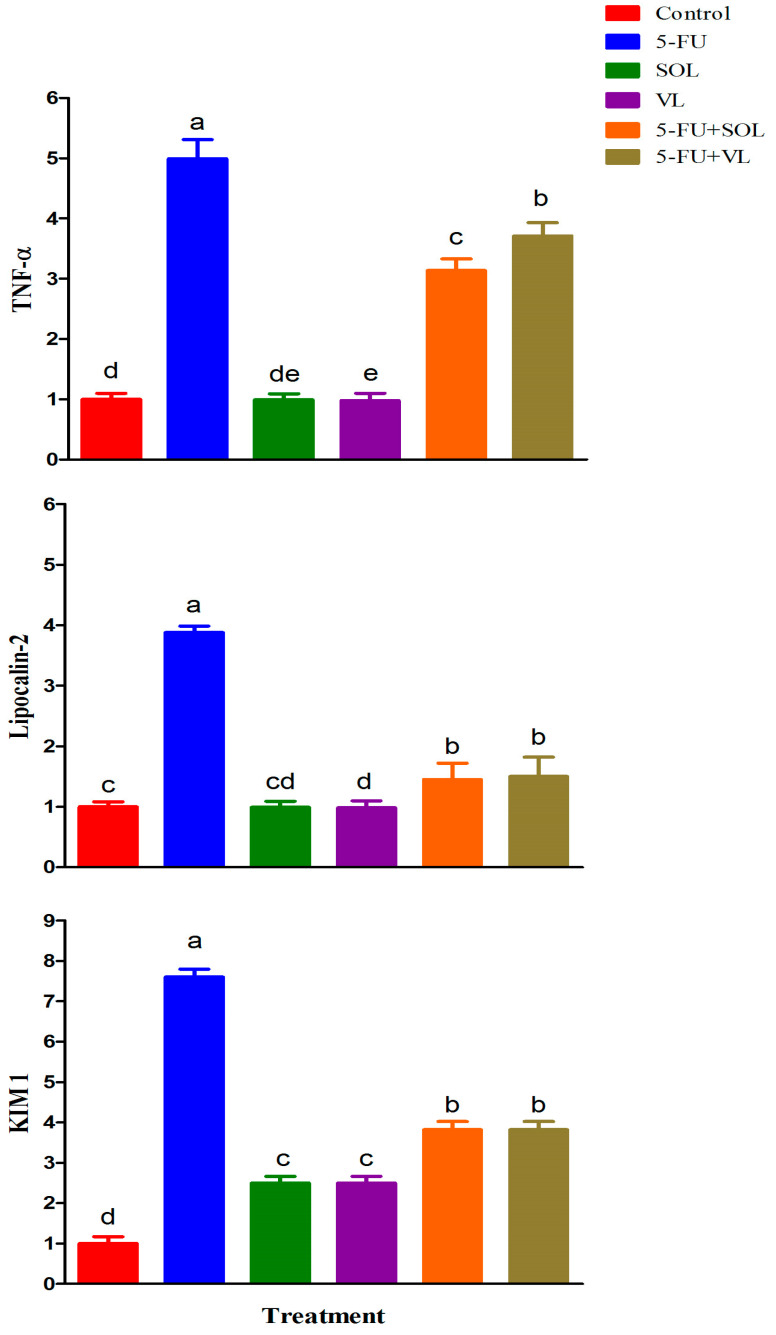
The effect of dietary treatments on gene expression of male rats. The expression of control is taken as 1.0. Data represented as the mean ± SE. ^a–e^ Means with different letters varied at *p* < 0.01.

The mRNA expression of the candidate genes is presented in Figure 1. 5-FU induced a significant increase in the relative expression of *TNF-α*, *lipocalin-2*, and *KIM 1* compared to the control ones (*p* < 0.05). Furthermore, the administration of SOL, VL, SOL+5-FU, and VL+5-FU induced significant decreases (*p* < 0.01) in the relative expression of *TNF-α*, *lipocalin-2*, and *KIM 1* compared to the 5-FU group.

### 2.6. Histopathological Examination

**Figure 2:**
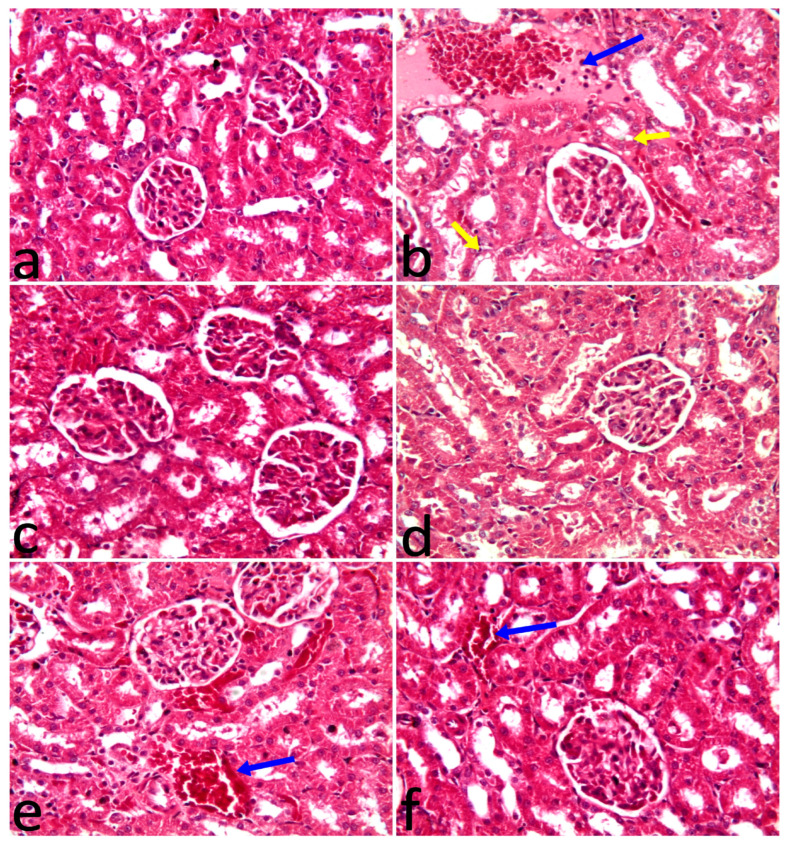
(**a**) The control group had normal renal structures like glomeruli and tubules. (**b**) The 5-FU group had homogenous faint pink fluid, congested blood vessels, inflammatory cell migration to the interstitial tissues among tubules (blue arrow), and vacuolation of the tubular epithelium (yellow arrows). (**c**) VL group had a normal shape of renal glomeruli and tubules. (**d**) *Saccharum officinarum* group showing a normal shape of renal structures. (**e**) 5-FU group plus chlorella vulgaris had only congestion of blood vessels (arrow). (**f**) Fluorouracil group plus SOL showing congestion of blood vessels (arrow). H&E X20.

The histopathological examination of the kidney is shown in Table 6 and Figure 2. All examined kidneys from the control, VL, and SOL groups revealed normal structures of the renal capsule, cortex, and medulla. Also, glomeruli, tubules, and blood vessels were of normal shape. The administration of 5-FU-induced congestion of blood vessels, interstitial edema, mild inflammatory cell infiltration, and mild tubular vacuolation. Administration of VL or SOL with 5-FU alleviated microscopic abnormalities in renal tissues.

## 3. Discussion

Gaining more knowledge on the effects of cancer therapy can help researchers create new cancer therapies and reduce the toxicities of anticancer medications. Crucially, not every cancer patient responds uniformly to treatments, and not every cancer patient has the same comorbidities or prior diseases. Owing to its effectiveness in treating a range of human cancers, 5-fluorouracil is a commonly used chemotherapeutic medication; yet, it has hepatotoxic and nephrotoxic adverse effects [16]. Apoptosis and oxidative stress are elevated in conjunction with this organ damage [17]. Consequently, the current study aimed to minimize 5-FU-induced nephrotoxicity using SOL and VL supplements.

Dissection of the animals used in this study revealed that the drop in body weight brought on by 5-FU may be due to fat and skeletal muscle loss [18], which agreed with those recorded by Safarpour et al. [19]. In the present study, SOL and VL lead to an increase in body weight parameters. This study supported Swinburn et al. [20], who observed that weight is generally gained through food intake, as noticed by increased body weight modification in the SOL rats. This may be due to energy acquired from the nutrients in the juice and the given feed. However, the findings of Ogunwole et al. [21] and Flavel et al. [22] observed a reduction in body weight after consuming *Saccharum officinarum* juice for a prolonged extent of time in rats, and that extract decreased body weight in mice fed high fat and high carbohydrate diets due to its abundant contents of polyphenols. The current study is in agreement with the research of Abd El Latif et al. [14], who observed that weight is generally gained through applied VL, which may be attributed to VL being a safe alga, and it is regarded as a superfood since it contains 60% protein, 18% amino acids, and 20% vitamins [12]. Moreover, VL is believed to be a microalga with nutritional potential that can improve certain physiological and biochemical processes for increased growth and immunity [23]. On the other hand, Alfaia et al. [24] reported a non-significant difference in body weight parameters in broiler chickens, which may be attributed to different animal species and different doses of VL.

Additionally, the administration of 5-FU significantly decreased Hb, PVC, RBCs, WBC count, and lymphocytes and significantly increased RDW and N/L ratio. Lymphocytes are immune cells basic in humoral and cellular immunity. Yahya et al. [25] concluded that 5-FU at a dose of 10 mg/kg day-by-day IP injection led to a decline in the hematological indices of rats. This is attributed to chemotherapy reducing the capacity of the bone marrow to create new ones, which lowers blood cell counts and the blood percentage of hemoglobin [26]. Our findings also agreed with Sharma et al. [27], who observed the detrimental effects of 5-FU on hematological parameters with a decrease in HB, RBCs, PCV, WBC count, and lymphocytes. Thus, we ameliorated the negative effects of 5-FU on hematological parameters by administration of SOL and VL. SOL is very rich in iron so it could improve blood parameters [28]. In addition, Wei et al. [29] and Abd El Latif et al. [14] reported the protective effect of VL on hematological parameters with enhancement in RBCs, PCV, Hb, WBC count, and lymphocytes. According to Magnadottir [30] and Khani et al. [31], white blood cells (WBCs) are immunological-competent immune system cells that are important against both infectious and non-infectious disorders. Fish-fed VL at varying doses showed an increase in the size of their WBC population. The beneficial effects of numerous VL components, such as vitamins and glucans, found in the VL cell wall, may contribute to this improvement. Thus, SOL and VL in our present experiment could improve the changed hematological parameters and regulate the untoward effect of 5-FU on blood parameters.

The findings of the present investigation proved that rats administrated 5-FU had impaired kidney functioning. The induced nephrotoxicity by 5-FU was confirmed by an increase in serum urea, creatinine, and uric acid concentration. Elevated levels of these parameters indicated that 5-FU-induced kidney damage [32,33]. In addition, hypoalbuminemia, or low serum albumin levels, is a strong independent predictor of acute renal damage [32,33]. In the current trial, SOL and VL significantly decreased the level of serum urea, creatinine, and uric acid, this finding was in line with previous research [14,34], respectively. Furthermore, SOL and VL administration could restore the serum levels of creatinine and urea to nearly normal values, protecting the kidneys from harm. Previous investigations used SOL and VL to treat oxidative stress [26,35]. These are consistent with the restorative effects of SOL and VL over serum clinical chemistry. Similarly, several studies found that administering SOL and VL prevented membrane fragility and had anti-inflammatory, antihypertensive, and antioxidant properties [36,37].

The present investigation observed that oxidative stress is a crucial process in 5-FU-caused kidney injury as verified by obvious declines in kidney SOD, GSH, and CAT and increases in MDA and NO concentrations. This finding aligns with recorded decreases in kidney antioxidants in the 5-FU group [38,39]. Intriguingly, MDA is the main reactive aldehyde produced during lipid oxidation. Accordingly, with the incidence of kidney damage, its kidney level is commonly used as a reliable indicator of lipid peroxidation [40]. Kidney damage noticed in the 5-FU-treated rats was supported by promoted MDA. Nevertheless, the decreased oxidative stress in the kidneys of SOL and VL- and VL-treated rats was due to lower NO and MDA values, indicating that SOL and VL augmented their antioxidant capacities by scavenging ROS induced by 5-FU [15,34]. NO is included in controlling numerous physiological processes. iNOS facilitates the production of NO that causes the release of peroxynitrite, which in turn prompts DNA damage and cell injury [41]. The present trial observed the antioxidant activity of SOL and VL through the rise in kidney SOD, CAT, and GSH as recorded previously by Mohammed et al. [42] and Yu et al. [43], respectively. This may be due to the detected capability of SOL and VL to improve antioxidant processes, leading to the scavenging of free radicals to permit kidney tissue restoration. The phenolic compounds identified in VL are responsible for its protective effects and antioxidant activity [44]. Additionally, it contains many trace elements required for the action of numerous antioxidant enzymes [45]. The present study was, therefore, aimed to estimate the antioxidant effect of SOL on 5-Fu-induced renal damage, which was linked to phytochemical components of SOL. SOL is an extremely abundant source of polyphenolics, primarily flavonoids, which are antioxidant components [46]. The antioxidant ability of VL has been related to its phenolic ingredients detected among other active phytoconstituents such as lutein, carotenoids, catechins, caffeic acid, gallic acid, chlorogenic acid, benzoic acid, and rutin [47]. It is generally recognized that oxidative stress and inflammation are closely related. Excessive ROS generation triggers a cellular signaling cascade that encourages the expression of proinflammatory cytokines as tumor necrosis factor-alpha (*TNF-α*), which has a critical role in the development of numerous illnesses [48]. Our findings verified that 5-FU administration elevated the gene expression of renal *TNF-α*, which agrees with the results of Ansari et al. [33]. On the other hand, our data revealed that treatment with SOL and VL is a powerful approach against 5-FU-induced inflammation. Additionally, VL reveals a potent anti-inflammatory agent via regulating inflammatory cytokine release as *TNF-α*. These findings are in harmony with the results of Abdelhamid et al. [49], who reported that VL administration downregulated *TNF-α* in splenic fish. Furthermore, even though they downregulate inflammatory mediators, VLs may be able to alter cellular antioxidant indicators, which could explain why they reduce inflammatory reactions [45]. Moreover, the present data disclosed that 5-FU induced obvious renal damage that was accompanied by upregulation of *KIM 1* and lipocalin-2 genes, which confirmed kidney damage and kidney function alteration. Previous studies have confirmed that *KIM 1* is a glycoprotein released by the proximal tubular cells and is known to be an early, accurate, and precise urine biomarker of renal damage [50]. *KIM 1* levels typically rise when the kidneys are damaged by toxins [51]. In this study, the nephrotoxic effect of 5-FU through increasing the levels of *KIM 1* agrees with Ali et al. [32], who reported that 5-FU increased the level of the *KIM 1* gene. An additional biomarker of kidney damage is lipocalin 2, another name for lipocalin-2 is neutrophil gelatinase-associated lipocalin (*NGAL*), a new 198-aminocytokine. Lipocalin 2 is a circulatory protein that, upon binding to megalin/glycoprotein and *GP330 SLC22A17* or *24p3R lipocalin 2 receptors*, is accountable for the transportation of tiny and hydrophobic molecules (hormones, prostaglandins, steroids, and free fatty acids) to target organs. Lipocalin 2 has been employed as a biomarker for acute and chronic renal damage [52]. In this study, the nephrotoxic effect of 5-FU through increasing the levels of lipocalin 2 agrees with Ali et al. [32], who reported that 5-FU increased the lipocalin 2 gene expression. Conversely, supplementation of SOL and VL can significantly downregulate the gene expression of *KIM 1* and lipocalin-2 in 5-FU-treated rats, indicating that SOL and VL can improve the renal function, encourage the healing of renal epithelial cells and the glomerular filtration rate, and augment kidney detoxification. Due to the beneficial effects of SOL and VL as potent anti-inflammatory and antioxidant agents, it could be used to minimize kidney toxicity induced by 5-FU. Additional research studies are needed to discover additional molecular mechanisms.

The findings of the histopathological assessment of kidneys from the control, SOL, and VL groups showed normal structures of the renal parenchyma. On the other hand, the administration of 5-FU caused structural changes including congestion of blood vessels, interstitial edema, mild inflammatory cell infiltration, and mild tubular vacuolation, our results agreed with [32,33]. 5-FU is an effective chemotherapeutic agent of antimetabolites. Nevertheless, its robust harmfulness limits its clinical application [53]. 5-FU-induced nephrotoxicity may be explained by inflammation response and oxidative stress to renal tissues [54]. Inflammatory reaction plays a major role in the incidence of renal damage. A complicated network of interactions between parenchymal cells and local immune cells, the recruitment of circulating immune cells, establishes an inflammatory reaction, a situation strictly associated with renal disease [55]. Moreover, the release of ROS due to oxidative conditions could contribute to renal tissue damage and alteration induced by 5-FU [17,56]. 5-FU caused oxidative stress as demonstrated by overstimulation of inflammatory cytokines and renal oxidative enzymes. In addition, the histopathological results revealed that SOL or VL with 5-FU reduced microscopic abnormalities in renal tissues caused by 5-FU alone. Thus, the current study aimed to minimize kidney toxicity induced by 5-FU following SOL and VL supplements. The reno-protective impact of SOL may be attributed to its high content of antioxidant constituents. Flavonoids and polyphenolic composites such as luteolin, apigenin, and tricin were presented in the maximum quantities. It has been suggested that tricin, luteolin, and apigenin work synergistically or additively in SOL, resulting in renal tissue that is almost normal in terms of histomorphology [23]. Moreover, VL has a high level of α and β-carotene, which react with various ROS and interfere with lipid oxidation and cellular partitions, it alleviates renal damage and pathological lesions in renal tissues [57].

## 4. Materials and Methods

### 4.1. Studied Materials

#### 4.1.1. 5-Fluorouracil

It was obtained as Utoral^®^ one vial (10 mL), which contains 500 mg fluorouracil (Hikma Specialized Pharmaceuticals, Badr City, Cairo, Egypt).

#### 4.1.2. *Saccharum officinarum* L. (SOL) and *Chlorella vulgaris* (VL)

Sugarcane juice was performed as illustrated by Khan et al. [44]. Bottles were kept in the refrigerator at 3–4 °C and used fresh after direct preparation. VL was obtained as pure powder taken from the Algal Biotechnology Unit, National Research Centre (Giza, Egypt). The required daily dose of VL is dissolved in water in suspension form on the day of administration to rats using an ultrasonic homogenizer (Biologics Inc., Manassas, VA, US) according to Abd El Latif et al. [14]. The total phenolic content of SOL and VL was performed using the Folin-Ciocalteu method following Al-Farsi et al. [58]. The total flavonoid content of SOL and VL was performed using the method of Kim et al. [59]. Analyses of phenolic and flavonoid compounds of SOL juice and VL extract were performed using HPLC apparatus (Agilent Series 1100, Agilent, VA, USA) according to the methods of Lin et al. [60] and Kuntic et al. [61]. The HPLC apparatus consisted of an auto-sampling injector, solvent degasser, two LC-pumps (series 1100), ChemStation software (version 11), and UV/Vis detector (set at 250 nm for phenolic acids and 360 nm for flavonoids). The analysis was achieved C18 column (125 mm × 4.60 mm, 5 µm particle size). Phenolic acids were separated by employing a gradient mobile phase of two solvents—Solvent A (methanol) and Solvent B (acetic acid in water; 1:25). The gradient program was begun with 100% B and was held at this concentration for the first 3 min. This was followed by 50% eluent A for the next 5 min, after which the concentration of A was increased to 80% for the next 2 min and then reduced to 50% again for the following 5 min detection wavelength at 250 nm. Flavonoids were separated by employing a mobile phase of two solvents: acetonitrile (A) and 0.2% (*v*/*v*) aqueous formic acid (B) with an isocratic elution (70:30) program. The solvent flow rate was 1 mL/min, and the separation was performed at 25 °C. The injection volumes were 25 μL. The phenolic and flavonoid components of SOL and VL are presented in Table 7 and Table 8, respectively.

### 4.2. Rats and Trial Design

Forty-eight healthy male albino Wistar rats, weighing from 100 to 120 g, were purchased from Laboratory Animal Colony (Giza, Egypt). The rats were maintained in the animal shelter of the College of Veterinary Medicine of Sadat City in well-ventilated cages, at a temperature of 25 ± 2 °C and 12 h light/dark. Animals were fed a balanced pellet diet with limitless access to water. The adaptation period was 2 weeks before the start of the investigation. Rats were weighed at the start of the trial (initial weight). The experimental protocols met the Guidelines for Animal Experimentation and were approved by the Ethical Committee of the Faculty of Veterinary Medicine, University of Sadat City, Egypt. Animal handling was carried out according to recommendations and under the regulations of Animal Care House (approval No, VUSC-006-1-24). The efficacy of SOL and VL against acute renal toxicity with 5-fluorouracil was determined using a protocol following Ali et al. [32]. Animals were randomly allocated into 6 groups of 8 rats each as illustrated in Table 9. The experimental length was 15 days.

Group 1, the control negative group, received 1 mL of distilled water orally per os for fifteen days, and on the 8th day, intraperitoneally (IP) injection with saline was performed. In group 2, the control positive group, the rats were treated with IP injection of 5-fluorouracil (150 mg/kg b.wt) on the 8th day [62]. Group 3, the SOL group, received *Saccharum officinarum* L. (sugarcane juice) orally (15 mL/kg b.wt.) daily for 15 days [34]. Group 4, the VL group, was given *Chlorella vulgaris* suspension orally in a dose of 400 mg/kg b.wt. daily for 15 days [63]. Group 5, the 5-FU+SOL group, was administered *Saccharum officinarum* orally in a dose of 15 mL/kg b.wt. daily for 15 days with an IP injection of 5-fluorouracil in a dose of 150 mg/kg b.wt. on the 8th day. Group 6, the 5-FU+VL group, received VL orally in a dose of 400 mg/kg b.wt. daily for 15 days with an IP injection of 5-fluorouracil in a dose of 150 mg/kg b.wt. on the 8th day.

### 4.3. Sampling

At the end of the investigation, rats were fasted overnight for blood sample collection. Blood samples were taken from five rats through retro-orbital bleeding under diethyl ether anesthesia (Sigma Chem. Co., St. Louis, MI, USA), and each blood sample was partitioned into two sections. The first one was kept in a vial containing EDTA for hematological studies. The other blood samples were gathered in non-heparinized centrifuge tubes and leftward to coagulate, then centrifuged (3000 rpm for 15 min) to obtain serum, and subsequently kept in a freezer (−20 °C) till further biochemical evaluation. After blood samples were collected, rats were sacrificed by cervical decapitation for kidney tissue sampling. The kidney specimens from each rat were immediately separated, bathed with cold saline to eliminate blood, and then dried on filter paper. Each kidney tissue sample was distributed into two portions. A part was stored at −80 °C for further biochemical and gene expression analyses. The other part was fixed in a 10% neutral formalin solution for subsequent histopathological examination.

### 4.4. Absolute and Relative Body and Organ Weights

Rats included in the experiment were weighed using a weight measurement scale before beginning treatment and on scarification day, and body weight gain was then calculated. Upon being sacrificed, the kidney was carefully removed, cleaned of any extraneous tissue, and weighed. The relative weights of kidneys (ROW) were calculated as follows [64].

ROW = [Absolute organ weight (g)/body weight of rat (g)] × 100.

### 4.5. Hematological Analysis

The blood samples were used immediately upon collection to estimate the following hematological parameters: total leucocyte count (TLC), differential leukocyte counts, platelet counts (Plt), hemoglobin (Hb) concentration, hematocrit value (PCV%), and red blood cells (RBCs) using automated hematology analyzer and blood cell counter (Sysmex F-800, Tokyo, Japan) [65].

### 4.6. Biochemical Assay

The renal tissue biomarkers were determined in the serum samples according to the manufacturer’s protocol (Biomed Company, Cairo, Egypt). Total proteins (TP) and albumin (Alb) were assessed according to Henry et al. [66]. Renal products such as creatinine [67] uric acid [68], and urea [69,70] were also measured.

### 4.7. Oxidant/Antioxidant Biomarkers in Tissue Homogenate

A part of kidney tissue was homogenized using a glass homogenizer with ice-cooled saline to make 25% *W*/*V* homogenate and then centrifuged (1700 rpm for 10 min). The supernatant was kept at –80 °C until further assay. Oxidative stability and antioxidant capability were determined colorimetrically using kits (Biodiagnostic, Dokki, Giza, Egypt) according to the manufacturer’s protocols. Renal Nitric Oxide Radical Scavenging Assay (NO) [71], glutathione peroxidase (GSH) [72], superoxide dismutase (SOD) [73], catalase activity [74], and malondialdehyde (MDA), the key reliable marker of peroxidation [75], were measured.

### 4.8. Inflammation and Kidney Injury Marker Genes (Quantitative RT-PCR)

RNA was extracted from the renal tissue using the Qiagen RNeasy Plus Mini kit (Qiagen, Hilden, Germany, GmbH). The concentration and purity of the RNA samples were measured by NanoDrop ND-1000 Spectrophotometer (NanoDrop Technologies, Wilmington, DE, USA) with absorption ratios of 260 and 260/280 nm. A 2 µg of highly purified RNA samples (with absorbance ratio 260/280~2) were reverse transcribed to prepare cDNA using SuperScript III reverse transcriptase kit (Invitrogen, Carlsbad, CA, USA) using the manufacturer’s instruction and protocol. Amplification was performed using Thermo Scientific Maxima^®^ SYBR Green/ROXqPCR Master Mix (2×) and Rotor-Gene Q Real-Time PCR System (Qiagen, MD, USA). Specific PCR primers for inflammation and kidney injury marker genes (Table 10) were blasted on NCBI/Blast to confirm their specificity for a required gene. Real-time PCR cycling conditions were 94 °C for 1 min one cycle of initial denaturation followed by 40 cycles of amplification (denaturation 10 s at 94 °C, annealing 30 s at 60 °C, and extension 60 s at 72 °C). Threshold Cycle (Ct) value, normalization for variation in the expression of TNF-α, lipocalin-2, and KIM-1 genes was performed representing the mean critical threshold value of the β-actin housekeeping gene. The quantitative relative expression of genes is determined by the 2^−∆∆Ct^ equation method according to Livak and Schmittgen [76].

### 4.9. Histopathological Examination

Kidney tissues from different groups were examined grossly and collected in 10% neutral buffered formalin for 3 days. Well-preserved tissues were trimmed, washed, routinely changed in different alcohol solutions, embedded into paraffin wax that was cut into 4-µm sections, and finally stained with hematoxylin and eosin stain [81]. Histologically, a scoring of renal abnormalities was performed semi-quantitatively as presented; congestion of blood vessels, edema, inflammation, and vacuolation were recorded as follows: (-): normal; (*): mild < 25%; (**): moderate < 50%; and (***): severe > 50% of examined sections.

### 4.10. Statistical Analysis

Data were subjected to one-way ANOVA (SPSS, version 21) followed by Duncan’s test to compare means at a probability of *p* < 0.05 [82]. Data are shown as the means ± SE.

## 5. Conclusions

In summary, SOL or VL decreased inflammation, oxidative stress, and kidney injury induced by 5-FU, which is used in cancer therapy. The treatment of SOL or VL decreased inflammation in kidney tissue by downregulating the expression of inflammatory marker genes, such as *TNF-α*, *lipocalin-2*, and *KIM 1*, which were all elevated by 5-FU. Subsequently, SOL or VL may be a successful supplement to prove the clinical use of 5-FU in treatments of different tumors with lessened renal toxicity.

## Figures and Tables

**Table 1:** Effect of SOL and VL alone or with 5-FU on body weight parameters.

	Treatment						
Items	Control	5-FU	SOL	VL	5-FU+SOL	5-FU+VL	*p*-Value
Initial BW, g	120 ± 1.91 ^b^	120 ± 4.76 ^b^	135 ± 2.92 ^a^	125 ± 4.04 ^ab^	117 ± 4.13 ^b^	122.5 ± 4.74 ^ab^	0.86
Final BW, g	156 ± 3.12 ^ab^	150 ± 4.37 ^b^	175 ±7.47 ^a^	174 ± 7.46 ^a^	154 ± 5.44 ^ab^	164 ± 12.19 ^ab^	0.04
Feed intake, g	148 ± 0.11 ^d^	151 ± 0.010 ^c^	156 ± 0.02 ^b^	179 ± 0.1 ^a^	135 ± 0.03 ^e^	151 ± 0.01 ^c^	0.001
Body gain, g	36.00 ±1.76 ^b^	30.00 ± 0.55 ^c^	40.00 ±4.78 ^ab^	49.00 ± 4.24 ^a^	37.00 ± 2.67 ^b^	42.5 ± 7.95 ^ab^	0.009
Feed conversion	4.11 ± 0.29 ^b^	5.03 ± 0.06 ^c^	3.9 ± 0.64 ^ab^	3.65 ± 0.30 ^a^	3.64 ± 0.55 ^a^	3.55 ± 1.20 ^a^	0.04

^a–e^ Means with no shared letters in the same row vary significantly (*p* < 0.05).

**Table 2:** Effect of SOL and VL alone or with 5-FU on absolute and relative kidney weight of the rats.

	Treatment						
Parameters	Control	5-FU	SOL	VL	5-FU+SOL	5-FU+VL	*p*-Value
Absolute kidney weight (g)	1.24 ± 0.09	1.31 ± 0.11	1.43 ± 0.05	1.28 ± 0.07	1.28 ± 0.01	1.48 ± 0.08	0.068
Relative kidney weight (g)	0.79 ± 0.05	0.83 ± 0.06	0.79 ± 0.04	0.76 ± 0.03	0.89 ± 0.03	0.90 ± 0.05	0.083

**Table 3:** Effect of SOL and VL alone or with 5-FU on blood profile of male rats.

	Treatment						
Items	Control	5-FU	SOL	VL	5-FU+SOL	5-FU+VL	*p*-Value
RBCs (10^6^/μL)	6.22 ± 0.49 ^bc^	5.33 ± 0.27 ^d^	6.96 ± 0.04 ^ab^	7.61 ± 0.18 ^a^	6.31 ± 0.20 ^bc^	6.01 ± 0.11 ^c^	<0.001
Hb(g/dL)	12.77 ± 0.45 ^b^	10.00 ± 0.26 ^d^	14.00 ± 0.22 ^a^	14.64 ± 0.42 ^a^	12.44 ± 0.32 ^bc^	11.66 ± 0.10 ^bc^	<0.001
PCV (%)	37.54 ± 1.19 ^c^	31.36 ± 0.51 ^e^	40.54 ± 0.47 ^b^	44.86 ± 1.21 ^a^	38.38 ± 0.77 ^bc^	34.65 ± 0.63 ^cd^	<0.001
MCV (fl)	63.84 ± 1.00 ^a^	56.52 ± 1.01 ^b^	55.92 ± 0.72 ^b^	57.96 ± 0.89 ^b^	58.02 ± 2.10 ^b^	58.18 ± 0.09 ^b^	0.001
MCH (pg)	21.32 ± 0.66 ^a^	19.54 ± 0.17 ^b^	19.40 ± 0.13 ^b^	19.24 ± 0.15 ^b^	19.50 ± 0.16 ^b^	19.3 ± 0.19 ^b^	0.001
MCHC (g/dL)	34.14 ± 0.54 ^ab^	34.08 ± 0.32 ^ab^	34.56 ± 0.21 ^a^	32.94 ± 0.04 ^b^	32.62 ± 0.24 ^b^	33.44 ± 0.33 ^bc^	0.002
RDW (%)	15.92 ± 0.97 ^c^	20.80 ± 0.30 ^a^	18.08 ± 0.16 ^b^	18.72 ± 0.34 ^b^	19.28 ± 0.19 ^ab^	19.86 ± 0.36 ^ab^	<0.001
WBCs (10^3^/μL)	12.91 ± 0.84 ^ab^	6.56 ± 0.87 ^d^	16.37 ± 1.81 ^a^	14.58 ± 1.19 ^a^	14.17 ± 1.55 ^ab^	9.62 ± 2.17 ^bc^	0.001
Neutrophil (%)	3.00 ± 0.01 ^b^	14.60 ± 1.16 ^a^	6.60 ± 1.02 ^b^	8.40 ± 1.36 ^ab^	8.60 ± 2.80 ^ab^	4.40 ± 0.40 ^b^	0.04
Lymphocyte (%)	94.00 ± 0.01 ^a^	80.00 ± 2.25 ^c^	84.80 ± 1.59 ^b^	84.60 ± 2.20 ^b^	83.80 ± 3.91 ^bc^	88.60 ± 0.67 ^ab^	0.01
Monocyte (%)	1.00 ± 0.01 ^c^	3.40 ± 0.67 ^b^	6.20 ± 0.48 ^a^	5.40 ± 0.67 ^ab^	5.00 ± 0.89 ^ab^	4.00 ± 0.31 ^ab^	<0.001
Eosinophil (%)	1.00 ± 0.00 ^b^	1.00 ± 0.44 ^b^	2.40 ± 0.24 ^a^	2.00 ± 0.31 ^a^	1.60 ± 0.24 ^ab^	2.00 ± 0.00 ^a^	0.004
Basophil (%)	1.00 ± 0.00	1.00 ± 0.00	1.00 ± 0.00	1.00 ± 0.00	1.00 ± 0.00	1.00 ± 0.00	0.88
N/L ratio	0.03 ± 0.01 ^c^	0.18 ± 1.23 ^a^	0.08 ± 1.33 ^c^	0.1 ± 1.89 ^b^	0.1 ± 3.23 ^b^	0.05 ± 0.60 ^c^	0.02

^a–e^ Means with no shared letters in the same row vary significantly (*p* < 0.05).

**Table 4:** Effect of SOL and VL alone or with 5-FU on serum kidney function.

	Treatment						
Items	Control	5-FU	SOL	VL	5-FU+SOL	5-FU+VL	*p*-Value
Creatinine (mg/dL)	0.93 ± 0.05 ^b^	1.32 ± 0.16 ^a^	1.14 ± 0.09 ^ab^	0.84 ± 0.11 ^b^	1.18 ± 0.14 ^ab^	0.96 ± 0.06 ^b^	0.04
Uric acid (mg/dL)	3.22 ± 0.28 ^bc^	4.38 ± 0.35 ^a^	3.52 ± 0.43 ^abc^	2.63 ± 0.18 ^c^	3.78 ± 0.29 ^ab^	3.17 ± 0.21 ^bc^	0.01
Urea (mg/dL)	44.42 ± 2.38 ^b^	53.40 ± 3.94 ^a^	46.80 ± 4.96 ^b^	45.94 ± 4.08 ^b^	47.10 ± 2.46 ^b^	38.14 ± 3.45 ^c^	0.04
Total protein (mg/dL)	7.64 ± 0.17	7.5 ± 0.28	7.68 ± 0.21	8.21 ± 0.21	8.90 ± 0.90	8.32 ± 0.15	0.50
Albumin (mg/dL)	3.62 ± 0.06	3.23 ± 0.03	3.45 ± 0.04	3.75 ± 0.08	3.35 ± 0.20	3.45 ± 0.09	0.38

^a, b, c^ Means with no shared letters in the same row vary significantly (*p* < 0.05).

**Table 5:** Effect of SOL and VL alone or with 5-FU on kidney oxidant/antioxidant biomarkers.

	Treatment						
Items	Control	5-FU	SOL	VL	5-FU+SOL	5-FU+VL	*p*-Value
MDA (nmol/g)	3.26 ± 0.30 ^c^	11.05 ± 0.20 ^a^	2.87 ± 0.10 ^cd^	2.37 ± 0.21 ^d^	5.54 ± 0.21 ^b^	5.23 ± 0.35 ^b^	< 0.001
CAT (U/g)	128 ± 3.80 ^b^	47.14 ± 3.03 ^d^	151 ± 3.66 ^a^	159 ± 0.02 ^a^	102 ± 4.13 ^c^	121 ± 2.03 ^b^	<0.001
SOD (U/g)	27.83 ± 2.27 ^c^	11.24 ± 1.05 ^d^	35.69 ± 2.15 ^b^	44.46 ± 2.333 ^a^	24.78 ± 0.84 ^c^	28.38 ± 1.55 ^c^	<0.001
GSH (mg/g)	49.24 ± 2.33 ^bc^	18.08 ± 1.38 ^e^	51.23 ± 1.28 ^b^	57.93 ± 1.66 ^a^	38.63 ± 2.50 ^d^	45.36 ± 1.87 ^c^	<0001
NO (μmol/g)	3.73 ± 0.22 ^c^	11.77 ± 0.89 ^a^	4.18 ± 0.55 ^c^	3.43 ± 0.37 ^c^	6.44 ± 0.40 ^b^	4.15 ± 0.56 ^c^	<0.001

^a–e^ Means with no shared superscript in the same row vary significantly (*p* < 0.05). 5-FU = 5-fluorouracil, SOL = *Saccharum officinarum*, VL = *Chlorella vulgaris*, MDA = malondialdehyde, CAT catalase, SOD = superoxide dismutase, GSH = reduced glutathione, and NO = nitric oxide.

**Table 6:** Semi-quantitative scoring of renal changes in control, 5-FU-, SOL-, and VL-treated groups.

Histopathology	Control	5-FU	SOL	VL	SOL+5-FU	VL+5-FU
Congestion	-	***	-	-	*	**
Edema	-	**	-	-	-	-
Inflammation	-	**	-	-	-	-
Vacuolation	-	*	-	-	-	-

Histopathology scoring: congestion of blood vessels, edema, inflammation, and vacuolation L were recorded as follows: (-): normal; (*): mild < 25%; (**): moderate < 50%; and (***): severe > 50% of examined sections.

**Table 7:** HPLC fractions of phenolic and flavonoid contents of SOL.

Items	Concentration
Total phenolics	0.95 mg gallic acid equivalent/g
Phenols	µg/g
Catechol	8.43
Syringenic	7.56
*p*-coumaric	14.56
Caffeic	12.21
Gallic	6.5
Ferulic	12.22
Total flavonoids	0.70 mg quercetin equivalent/g
Flavonoids	µg/g
Quercetin	18.72
Kaempferol	16.60
Luteolin	8.27
Apigenin	13.41
Catechin	8.65

**Table 8:** HPLC fractions of phenolic and flavonoid contents of VL.

Item	Concentration
Total phenolics	38.34 mg gallic acid equivalent/g
Phenols	µg/g
Resorcinol	4.20
Chlorogenic	3.80
*p*-coumaric	14.49
Caffeic	13.65
Gallic	4.8
Ferulic	14.02
Total flavonoids	22.78 mg quercetin equivalent/g
Flavonoids	µg/g
Quercetin	12.50
Kaempferol	13.46
Rutin	3.46
Apigenin	10.6
Catechin	2.43
Hesperetin	12.64

**Table 9:** The experimental design.

	Days of Experiment		Days of Sacrifice 16th Day
Treatment Groups	On 1st to 7th day of the experiment	On the 8th day of the experiment	On 9th to 15th day of the experiment
Control	Distilled water 1 mL	Normal saline 1 mL	Distilled water 1 mL
5-FU	---------------	5-FU (150 mg/kg b.wt.)	---------------
SOL	SOL 15 mL	Normal saline	SOL 15 mL
VL	VL 400 mg/kg b.wt.	Normal saline	VL 400 mg/kg b.wt.
5-FU+SOL	SOL 15 mL	5-FU (150 mg/kg b.wt.)	SOL 15 mL
5-FU+VL	VL 400 mg/kg b.wt.	5-FU (150 mg/kg b.wt.)	VL 400 mg/kg b.wt.

**Table 10:** Sequence of TNF-α, lipocalin-2, KIM-1, and β-actin genes primers.

Gene	Forward Primer (5′-----3′)	Reverse Primer (5′-----3′)	References
*TNF-α*	GCATGATCCGCGACGTGGAA	AGATCCATGCCGTTGGCCAG	[77]
*Lipocalin-2*	GATTCGTCAGCTTTGCCAAGT	CATTGGTCGGTGGGAACAG	[78]
*KIM-1*	GGTCACCCTGTCACAATTCC	CTCGGCAACAATACAGACCA	[79]
*β-actin*	AGGAGTACGATGAGTCCGGC	CGCAGCTCAGTAACAGTCCG	[80]

## Data Availability

The data presented in this study are available on request from the corresponding author.
